# Selection and validation of reference genes for quantitative gene expression studies in *Erythroxylum coca*

**DOI:** 10.12688/f1000research.2-37.v1

**Published:** 2013-02-08

**Authors:** Teresa Docimo, Gregor W Schmidt, Katrin Luck, Sven K Delaney, John C D'Auria

**Affiliations:** 1 Max-Planck-Institut für Chemische Ökologie, Jena, D-07745, Germany; 2 Current address: Instituto Biologia e Biotecnologia Agraria (CNR), Milan, 20133, Italy; 3 Current address: Department of Biosystems Science and Engineering, Eidgenössische Technische Hochschule Zurich, Basel, 5048, Switzerland; 4 School of Biotechnology and Biomolecular Sciences, University of New South Wales, Sydney, 2052, Australia

## Abstract

Real-time quantitative PCR is a powerful technique for the investigation of comparative gene expression, but its accuracy and reliability depend on the reference genes used as internal standards. Only genes that show a high level of expression stability are suitable for use as reference genes, and these must be identified on a case-by-case basis.

*Erythroxylum coca* produces and accumulates high amounts of the pharmacologically active tropane alkaloid cocaine (especially in the leaves), and is an emerging model for the investigation of tropane alkaloid biosynthesis. The identification of stable internal reference genes for this species is important for its development as a model species, and would enable comparative analysis of candidate biosynthetic genes in the different tissues of the coca plant. In this study, we evaluated the expression stability of nine candidate reference genes in *E. coca *( *Ec6409*, *Ec10131*, *Ec11142*, *Actin*, *APT2*, *EF1α*, *TPB1*, *Pex4*, *Pp2aa3*). The expression of these genes was measured in seven tissues (flowers, stems, roots and four developmental leaf stages) and the stability of expression was assessed using three algorithms (geNorm, NormFinder and BestKeeper). From our results we conclude that *Ec10131* and *TPB1* are the most appropriate internal reference genes in leaves (where the majority of cocaine is produced), while *Ec10131 *and *Ec6409* are the most suitable internal reference genes across all of the tissues tested.

## Introduction

*Erythroxylum coca* has been cultivated by humans for more than 8000 years and has been selected for high-level production of cocaine, a pharmacologically active tropane alkaloid. Cocaine and other tropane alkaloids such as atropine and scopolamine act on the nervous system, and their activity is largely due to their common chemical backbone (the tropane nucleus) ^1^. Despite the socioeconomic importance of cocaine and other tropane alkaloids, the molecular basis for the biosynthesis of the tropane nucleus remains unknown. *E. coca* is emerging as a model for the investigation of tropane alkaloid synthesis ^2– 4^, and shows high-level, localized tropane alkaloid production and storage in its leaf tissue ^3, 4^.

We have performed metabolic and enzymatic studies to identify the molecular and biochemical basis of tropane alkaloid biosynthesis in *E. coca*, and have developed a number of genomic tools such as expressed sequence tag (EST) libraries and 454 sequence databases ^2– 4^. Quantitative real-time reverse-transcription PCR (qRT-PCR) would be a further source of information on candidate tropane alkaloid biosynthesis genes in the different tissues of the coca plant.

qRT-PCR is widely used to quantify and compare levels of gene transcription ^5^. Variables such as RNA quality and the efficiencies of reverse transcription and PCR may compromise the accuracy and reliability of qRT-PCR, and so results are typically ‘normalized’ by comparison with one or more internal reference genes ^6^. The internal reference genes must be stably expressed, and the most stable reference genes vary widely in different species, tissues and sets of experimental conditions. Therefore, the identification of stable reference genes is a crucial step in the design of qRT-PCR experiments.

Traditionally, ‘Housekeeping’ genes such as actin, glyceraldehyde 3-phosphate dehydrogenase (GAPDH) and ubiquitin were used for data normalization ^7, 8^. These genes were widely assumed to have a uniform level of expression due to their involvement in fundamental cellular processes. However, evaluation of the expression stability of classical housekeeping genes in many species including *Arabidopsis thaliana*, *Oryza sativa*, *Zea mays* and *Linum usitatissimus* ^9– 12^ has revealed unstable expression of these genes under a range of experimental conditions. In addition, several novel reference genes have been shown to be more stably expressed than classical housekeeping genes ^13^. Hence there is a need for systematic validation of internal reference genes in each organism and experiment ^9, 14^.

The stability of candidate internal reference genes may be assessed using a number of models, including geNorm ^15^, NormFinder ^16^ and BestKeeper ^17^. These models differ significantly in their assumptions, and so candidate genes are often assessed with several of these algorithms ^18^. geNorm iteratively calculates an expression stability value (M) for each candidate gene. This is based on the mean pairwise variation between the gene and the other candidate genes across all samples. Genes with lower M values are more stably expressed, and less stable genes (with higher M) are progressively excluded from the analysis. The optimal number of reference genes for qRT-PCR normalization may also be determined by identifying the smallest number of genes needed to minimize mean variation. By contrast, NormFinder estimates the standard deviation for each gene relative to the global expression of all genes included in the analysis, and genes with lower standard deviations are considered better reference genes. BestKeeper uses a third approach involving the calculation of a stability index (the ‘BestKeeper index’ or BKI), which is assumed to represent the highest level of stability because it includes all genes across all samples. The stability of each reference gene is assessed by its correlation with the BKI, with a high correlation indicating a more stable reference gene ^15– 17^.

In this study, we evaluate the stability of nine candidate reference genes ( *Ec6409*, *Ec10131*, *Ec11142*, *Actin*, *APT2*, *EF1α*, *TPB1*, *Pex4* and *Pp2aa3*) in a variety of *E. coca* tissues (four developmental leaf stages, stems, roots and flowers). We then identify the most stable internal reference genes using the geNorm, NormFinder and BestKeeper algorithms and present guidelines for transcript analysis in different tissues of *E. coca* by qRT-PCR.

## Materials and methods

### Plant material

*Erythroxylum coca* was obtained from the Bonn Botanical Garden. Plants were grown at 22°C under a photoperiod of 12 h light/12 h dark with relative humidities of 65% and 70% for light and dark conditions respectively (and fertilized once a week with Ferty 3 (15-10-15) and Wuxal Top N (Planta, Regenstauf, Germany).

**Figure 1.**
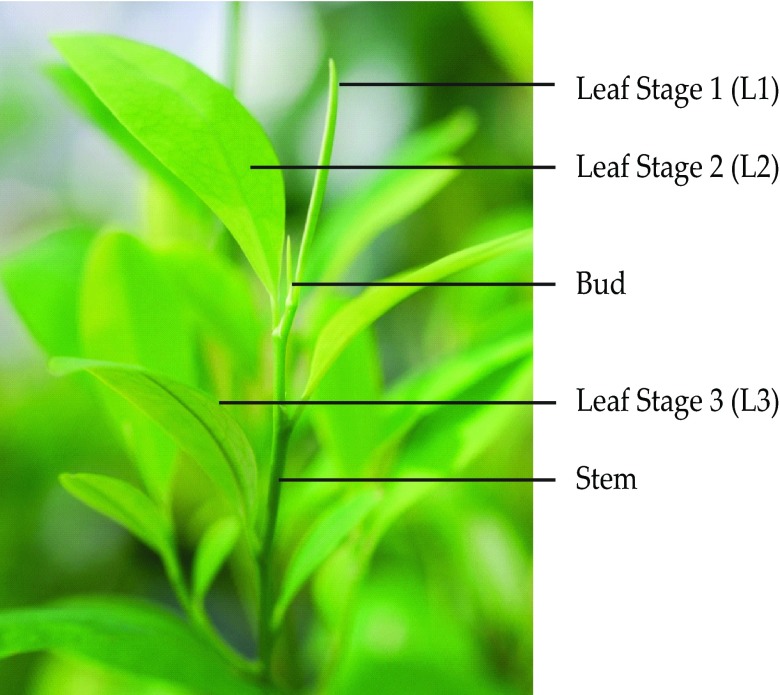
Developmental leaf stages of *Erythroxylum coca* plant. Leaf Stage I (L1) young rolled leaves, Leaf Stage II (L2) young expanded leaves, Leaf Stage III (L3) fully mature leaves.

The organs used for RNA extraction and qRT-PCR analysis were obtained from four-month old *E. coca* plants grown from rooted cuttings. Leaves in four developmental stages, roots, stems and flowers were analysed. The leaf developmental stages were: leaf buds; young expanding leaves in a rolled state (Stage 1); young expanded (unrolled) leaves (Stage 2); and fully mature leaves (Stage 3) (see Figure 1).

### RNA extraction and cDNA synthesis

Total RNA was extracted from 100 mg of fresh plant tissue using a total RNA extraction kit (Invitek, Berlin, Germany). Genomic DNA was removed by treatment with RNAse-free DNAse I (Qiagen, Hilden, Germany). RNA quality was assessed on an Agilent Bioanalyzer 2100 using a RNA 6000 Nano Kit (Agilent, Böblingen, Germany). RNA concentration was determined using a NanoDrop 2000 c spectrophotometer (NanoDrop Technologies, Wilmington, USA). cDNA was synthesized using a Super Script III First Strand Kit (Invitrogen, Karlsruhe, Germany) according to the manufacturer’s instructions. In brief, random hexamer primers and deoxyribonucleoside5’-triphosphates (dNTPs) were added to 5 µg total RNA and the mixture was incubated at 65°C for 5 min before brief chilling on ice. The first strand was then reverse transcribed by adding First Strand Buffer, 20 mM dithiothreitol and Super Script III reverse transcriptase to a final volume of 20 µl and incubating the mixture at 42°C for 1h. The resulting cDNA was diluted 1:20 (vol:vol) with deionized water and stored at -20°C.

### Reference gene selection

Candidate reference genes were selected from an *E. coca* 454 sequence library ^2^ based on their homology to previously reported reference genes in *A. thaliana* ^9^. Nine candidate reference genes with an E-value higher than 2e ^-72^ were identified by BlastN comparison as orthologues to *Arabidopsis* genes: Expressed protein (Ec6409), Expressed protein (Ec10131), Clathrin adaptor complex subunit (Ec11142), Actin (ACT), Adenine phosphoribosyl transferase 2 (APT2), Elongation factor 1 alpha (EF1α), Protein tyrosine phosphatase 1B (TPB1), Peroxin 4 (Pex4) and Pp2aa3-like protein. Primers for qRT-PCR were designed using Primer Express 3.0 (Applied Biosystems) and their sequences are shown in Table 1.

**Table 1.** Description of *Erythroxylum coca* candidate reference genes. GenBank accession numbers are given for each gene used in this study. The orthologous locus in *A. thaliana* is referred to by its AGI (Arabidopsis Genome Intitiative) designation. Similarity values are represented by E-values for the pairwise comparison of the coca gene with its Arabidopsis ortholog. PCR amplification efficiencies and the regression coefficients for their standard curves are reported for each primer pair.

Gene	Genbank accession number	Ortholog locus in *A. thaliana*	Similarity (E-value)	PCR efficiency	R ^2^ of standard curve	Primer sequence (forward/reverse)
*Actin*	JN020155	AT5G09810	2e ^-40^	97%	0.9974	GGATTTCCAAAGGTGAATACGATG/ TTGAACCAGCAAAGTTGAATAAGC
*APT2*	JN020149	AT5G11160	1e ^-16^	88%	0.9947	ACTCAGAGAGCGAGAGAGGATGTT/ TCAACTCCAGCAACCACAGAAATG
*EF1α*	JN020156	AT5G60390	0.00	84%	0.9981	TGGAGGTATTGACAAGCGTGTGATTGAGAG/ TTTGACACCAAGAGTGAAAGCAAGAAGAGC
*Ec11142*	JN020151	AT5G46630	2e ^-72^	83%	0.9967	ACATTACCAAAGCAGGCTCATACG/ TACATCTTCTCACCACCAACACAGG
*Ec10131*	JN020153	AT2G32170	8e ^-45^	79%	0.9916	TGGAAGGGTAGTGGGGTAACAATG/ GAGCGTAGTCGTCAGAGAAGGC
*Ec6409*	JN020150	AT4G26040	0.013	92%	0.9984	GAAGAGACAAGTGGTGGGGTGAG/ AGAAGAGAGCAAAGAGGAAGAGTGG
*Pp2aa3*	KC189827	AT1G13320	e ^-144^	88%	0.9860	TGCTCCTGTTATGGGTCCTGAAG/ TGCTCCTGTTATGGGTCCTGAAG
*Pex4*	JN020157	AT5G25760	4e ^-34^	88%	0.9968	GTCGGTTCTTTAGCAAGGTCAGTG/ CGTGGTGGCGGTGGTTGG
*TPB1*	JN020152	AT3G01150	e ^-104^	93%	0.9996	CCGATTGAAGCCATAACAGGAGAC/ CCCACAGGACCAGCACCAG

All primer pairs were validated prior to their use in gene expression analysis. PCR reactions were performed with each primer pair and the products were visualised by gel electrophoresis to confirm the presence of a single PCR product of the expected size. The sequence specificity of the PCR products was also verified by sequencing.

### Quantitative real-time PCR

All PCR reactions were performed on a Stratagene Mx3000P (La Jolla, USA). Each reaction contained 12.5 µl Brilliant Sybr Green (Agilent/Böblingen, Germany), 0.375 µl Rox, 0.4 µM primers and 1 µl cDNA in a final volume of 20 µl. All samples were run in triplicate. The thermocycling conditions were denaturation at 95°C for 10 min; followed by 40 cycles of denaturation (95°C, 15 s) and annealing/extension (60°C, 1 min). A melting curve analysis protocol was performed after completion of the PCR reaction to confirm the absence of multiple amplicons and/or primer dimers. A no template control (NTC) was included to ensure the absence of contamination. In addition, the presence of genomic DNA contamination was excluded by performing reactions without reverse transcriptase. PCR efficiency was determined using a standard curve based on between five and seven different four-fold dilutions of a cDNA cloned amplicon.

### Data analysis

Cycle threshold (Ct) values were exported from the MxPro software (Stratagene) to Microsoft Excel using the qBASE v1.3.5 macro ^19^. PCR efficiencies and regression coefficients were calculated in qBASE and are reported in Table 1. The expression stability of the nine reference genes in *E. coca* tissues was evaluated with geNorm v3.5 ^15^, NormFinder ^16^ and Bestkeeeper v1 ^17^. Relative expression quantities were exported from qBASE and analyzed in Microsoft Excel using the geNorm v3.5 and NormFinder macros. For analysis using the BestKeeper macro, Ct values from the MxPro Software and PCR efficiencies calculated by qBASE were utilized.

## Results

### Selection and expression profiling of candidate reference genes

**Supplementary Figure 1.**
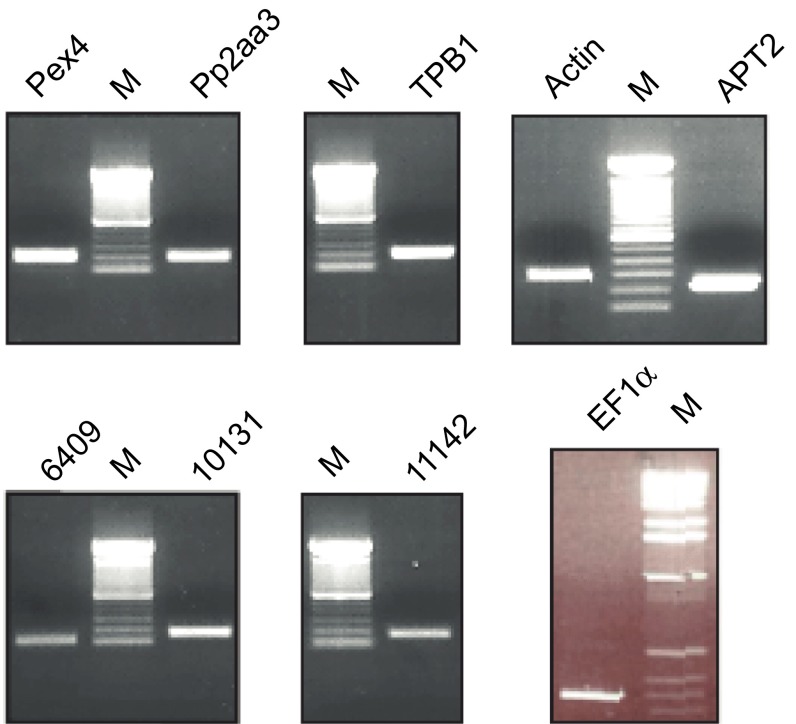
Specificity of qRT-PCR primers and amplicon length. The lane marked M represents a 1 Kb ladder (Invitrogen, California) used for size comparisons.

A similarity search (BlastN) between previously identified reference genes from *A. thaliana* ^9^ and an *E. coca* 454 sequence library ^2^ was conducted to identify orthologous sequences. Nine *E. coca* genes with high similarity to *A. thaliana* were selected and PCR primers targeting these sequences were developed (see Table 1). To confirm the specificity of the primers and identity of the amplicons, RT-PCR was performed on cDNA from four developmental leaf stages, stems, roots and flowers. Primer specificity was investigated by electrophoresis and a single amplicon of the expected size was obtained for each primer pair ( Supplementary Figure 1). Sequence analysis of ten cloned amplicons revealed that the amplified fragments were identical to the targeted sequences in the 454 sequence database. All primer pairs achieved amplification in fewer than 35 cycles in all samples, demonstrating that all of the candidate reference genes are expressed at experimentally useful levels. The ΔC _t_ between samples and no template controls (NTCs) was always greater than five cycles, showing that contamination during the setup of the experiment was negligible ^20^. All RNA samples were tested for contamination with genomic DNA by performing qPCR analysis on negative control reverse transcriptase reactions in which the reverse transcriptase was omitted. No amplification product could be detected in these control reactions.

The gene-specific amplification efficiency was calculated by linear regression analysis of the standard curve and ranged between 79% (Ec10131) and 97% (Actin). The coefficient of correlation ( *r* ^2^) of the linear regression analysis was always greater than 0.986 as shown in Table 1, indicating a linear relationship between C _t_ values and log-transformed transcript quantities in the range of the standard curve.

**Supplementary Figure 2.**
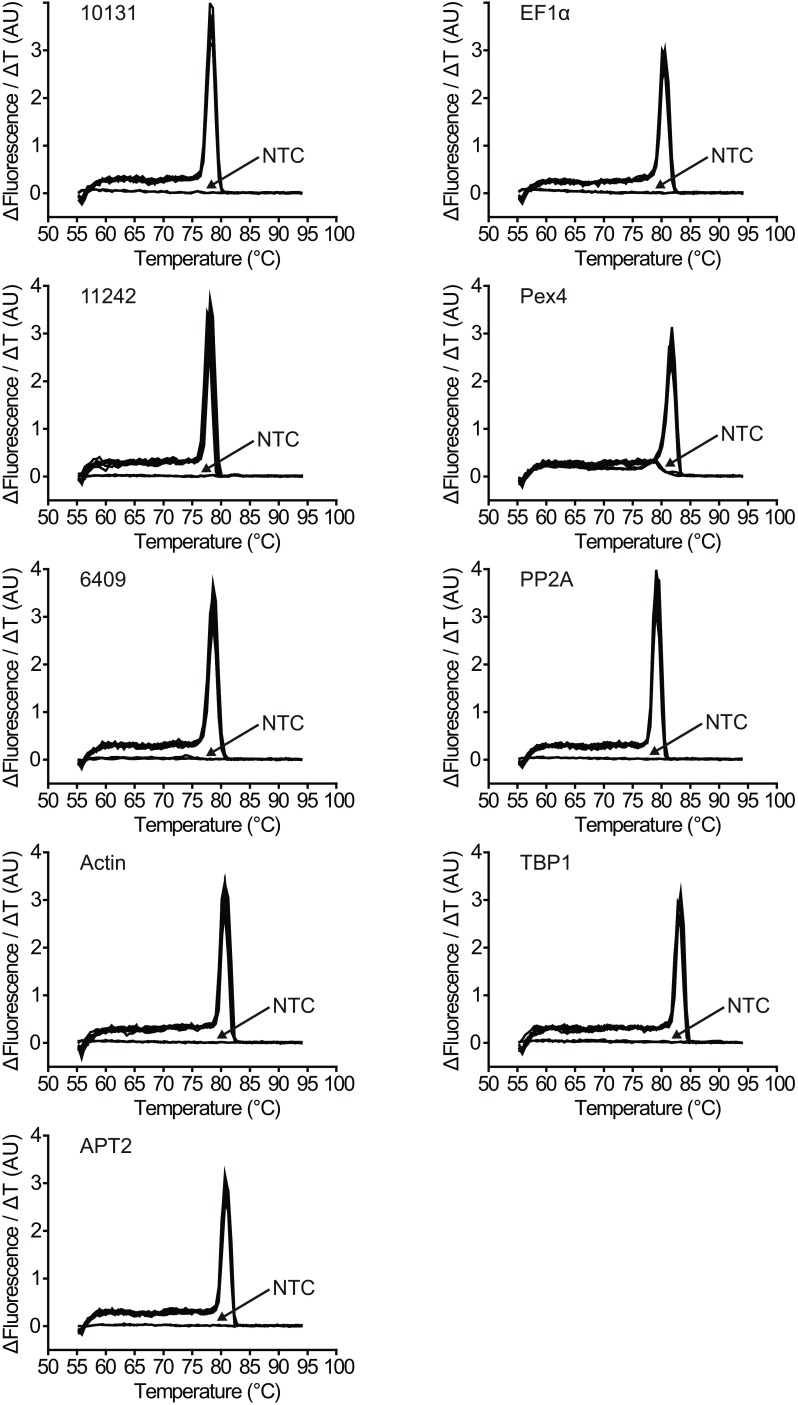
Melting curve analysis of RT-PCR products. NTC indicates: no template control.

To ensure that the primer pairs are specific for the desired sequence in all samples and do not target homologous transcripts in some sample subsets, a melting curve analysis of each sample was performed after PCR amplification ( Supplementary Figure 2). A single peak in the melting curve specific for each primer pair was obtained for all samples, and no peak could be observed in the melting curves of the control reactions (NTC and negative control reverse transcription reactions).

### Expression stability of candidate reference genes

The expression stability of the candidate genes were evaluated with the geNorm, NormFinder and BestKeeper algorithms ( Table 2). C _t_ values were transformed to relative quantities using qBASE prior to analysis with geNorm and NormFinder, while C _t_ values and PCR efficiencies were used in BestKeeper. The cDNA samples were considered as either a single, diverse set derived from all organ samples; or as two subsets derived from leaf buds and leaves (leaf buds, Stage 1, Stage 2 and Stage 3 leaves) or mature organs (Stage 3 leaves, flowers, roots and stems).

**Table 2.** Ranking of *Erythroxylum coca* reference gene stability in all *Erythroxylum coca* tissues according to the geNorm, BestKeeper and NormFinder algorithms.

Gene rank	geNorm (M*, V _n/n+1_)	BestKeeper (correlation coefficient, *r*)	NormFinder (stability value)
1	Ec10131/6409 (0.28)	Actin (0.784)	Pp2aa3 (0.291)
2		Ec6409 (0.768)	Ec6409 (0.294)
3	Pp2aa3 (0.30; 0.095)	APT2 (0.765)	Ec11142 (0.300)
4	TPB1 (0.34; 0.083)	Pp2aa3 (0.737)	EF1α (0.304)
5	Ec11142 (0.38; 0.080)	EF1α (0.73)	TPB1 (0.339)
6	EF1α (0.50; 0.115)	Pex4 (0.715)	Ec10131 (0.350)
7	Actin (0.62; 0.125)	TPB1 (0.688)	Actin (0.483)
8	APT2 (0.72; 0.120)	Ec11142 (0.661)	APT2 (0.596)
9	Pex4 (0.88; 0.147)	Ec10131 (0.638)	Pex4 (0.904)

*M indicates stability values listed from most stable to least stable.

geNorm calculates the average expression stability value (M) for each candidate gene on the basis of the average pair-wise variation between all genes analyzed. geNorm analysis indicated that *Ec10131* and *Ec6409* are the most stable candidate reference genes across all of the *E. coca* tissues tested ( Table 2). In the leaf bud/leaf sample subset, *Ec10131*, *TPB1* and *Ec6409* were ranked as the three most stable genes (in that order) ( Supplementary Table 1), while in the mature organ subset *Ec10131* and *Ec6409* were again ranked as the most stable. In contrast, *Pex4* and *APT2* were consistently ranked as the least stable in all sample subsets ( Table 2 and Supplementary Table 1 and Supplementary Table 2). The ‘housekeeping’ genes *Actin* and *EF1α* were relatively unstable and were ranked at positions six and seven (respectively) in all sample sets.

The optimal number of reference genes required for accurate normalization in the respective sample sets (all samples, leaf bud/leaf and mature tissues) was determined by calculating the mean variation in each normalisation factor (V) and then observing the effect of iterative addition of the next most stable reference gene (V _n_/V _n+1_) (as detailed in Vandesompele *et al.* 2002 ^15^). In each case, the two most stable reference genes were sufficient for accurate normalization, since inclusion of a third gene had little impact on the calculation of the normalization factor (V _n_/V _n+1_ below 0.15).

BestKeeper ranks gene stability by calculating the correlation coefficient ( *r*) between the expression of each candidate gene and the BestKeeper index (BKI; calculated using all genes across all samples). Across all of the samples tested, BestKeeper indicated that *Actin* ( *r* = 0.784) and *Ec6409* ( *r* = 0.768) were the most stable, while *Ec10131* was ranked as the least stable ( *r* = 0.638). In the leaf bud/leaf sample subset, *Actin* ( *r* = 0.869) and *APT2* ( *r* = 0.868) had the highest correlation with the BKI, and *Ec10131* again showed the lowest correlation ( *r* = 0.385). In the mature organs sample subset, *Pex4* and *APT2* were strongly correlated with the BestKeeper index ( *r* = 0.767 and *r* = 0.724, respectively), whereas *Ec10131* showed low correlation ( *r* = 0.309) ( Supplementary Table 1 and Supplementary Table 2).

To provide a further ranking of gene stability, the results were also evaluated with NormFinder, in which candidate reference genes are ranked according the variance of their expression relative to the expression variance within a defined group of samples ^16^. *Pp2aa3* was the most stably expressed gene with the lowest expression variance (stability value of 0.291), followed by *Ec6409* and *Ec11142*, when all samples were included in the calculation. When the leaf bud/leaf and mature organ subsets of samples were considered, the rankings varied considerably ( Supplementary Table 1 and Supplementary Table 2). *Actin*, *APT2* and *Pex4* were always ranked as the seventh, eighth and ninth most stable reference genes (respectively), but there was no consistent order of ranking for the other reference genes. The NormFinder rankings were also distinct from the geNorm rankings, although both algorithms identified *Actin*, *APT2* and *Pex4* as having the least stable expression profiles.

Raw Ct values and relative quantities for Erythroxylum coca reference genes Raw data based on Ct values obtained for each gene used in this study is provided. In addition, the calculated relative quantities for each gene tested in the different plant tissues is supplied as a separate file. NTC = Non template control (H20 control); STD = Standard for standard curve analysis; UNKN = Unknown (i.e. sample to quantify)

## Discussion

Real time RT-PCR has become a central technique for the evaluation of quantitative changes in gene expression ^21– 24^. Reliable and accurate expression data can only be obtained by normalization with stably expressed reference genes. Normalization is an essential prerequisite for the correct measurement of gene expression changes in different plant tissues, organs, developmental stages or treatments of a given plant species and is highly influenced by the choice of reference genes. Traditional reference genes (e.g. actin and ubiquitin) are useful as stable reference genes in some experiments ^9, 25^, but their expression is often highly variable ^26– 28^, and is often inferior to the stability of less-commonly used genes ^8^. Therefore it is important to assess the expression stability of several candidate reference genes before gene expression studies are performed. Several models including geNorm, NormFinder and BestKeeper have been developed to rank candidate reference genes on the basis of their expression stability. These methods often vary in their stability rankings ^18, 25^ and so expression data is commonly analysed using several approaches.

In this study, we report the identification and validation of nine candidate reference genes in *E. coca* ( *Ec6409*, *Ec10131*, *Ec11142 Actin*, *APT2*, *EF1α*, *TPB1*, *Pex4* and *Pp2aa3*). These genes were identified by analysing a 454 *E. coca* sequence library for sequences with homology to the top 100 reference genes of Arabidopsis ^9^, on the assumption that homologous genes are likely to have similar expression patterns. Primer pairs specifically targeting the *E. coca* transcripts were successfully developed and evaluated: all primer pairs produced only the expected amplicon and were highly efficient ( Table 1 and Supplementary Figure 1). The relative stabilities of the candidate reference genes were then assessed using geNorm, BestKeeper and NormFinder ( Table 2 and Supplementary Table 1 and Supplementary Table 2).

geNorm produced similar results in all sample sets. *Ec6409* and *Ec10131* were always identified as two of the three most stably expressed reference genes (although *Ec10131* and *TPB1* were most stable in the leaf bud/leaf sample subset), and *Actin*, *APT2* and *Pex4* were always identified as the least stable. geNorm may identify co-regulated genes as stable reference genes ^16^. However, exclusion of either *Ec10131* or *Ec6409* did not change the gene rankings (not shown), suggesting that their high ranking is not attributable to co-regulation.

BestKeeper yielded very different rankings to geNorm, and these varied according to the sample subset. The inconsistent results with BestKeeper may be explained by several features of the BestKeeper algorithm. Calculation of the BestKeeper index excludes genes with a standard deviation of more than one C _t_ value, which results in the exclusion of different genes in different sample sets ^17^. Extensive variation in C _t_ values is to be expected in a non-normalized data set, and so the algorithm may not be able to effectively distinguish between stable and unstable reference genes. In our experiments, the candidate *E. coca* reference genes showed very similar correlations with the BestKeeper index, suggesting that the algorithm could not distinguish between the genes to produce useful stability rankings. NormFinder produced a third ranking of gene stability that differed from both BestKeeper and geNorm. *Pp2aa3* and *Ec6409* were ranked as the most stably expressed genes when all samples were considered ( Table 2). geNorm also identified *Ec6409* as one of the most stable genes in the entire sample set. However, only *Pp2aa3* was consistently ranked by Normfinder, geNorm and BestKeeper as one of the most stable genes in the leaf bud/leaf and mature organs sample sets, and there was no consistency between the algorithms in the order of ranking for the most stable genes ( Table 2). The ranking of the least stable genes was more consistent: NormFinder identified *Actin*, *APT2* and *Pex4* as the least stable genes in all of the sample sets, and geNorm ranked these genes in the same order.

The NormFinder, BestKeeper and geNorm models have been shown to produce conflicting stability rankings in many studies ^18, 29^. The rankings produced by one or more of the models may be combined to produce a hybrid ranking ^18^, but this complicates the analysis by merging models with very different underlying assumptions. Hence, we favour using a single model when possible.

geNorm produced a consistent gene ranking across all of our samples, and provides a clear rationale for determining the minimum number of genes required for accurate normalization. We therefore recommend the use of *Ec10131* and *Ec6409* as internal reference genes for most *E. coca* sample sets. If leaves and leaf buds are the primary organs of interest, then we recommend the use of *Ec10131* and *TPB1*. These results provide a foundation for qRT-PCR studies in *E. coca*, and will further its development as a model of tropane alkaloid biosynthesis.

## Figures and Tables

**Supplementary Table 1.** Ranking of *Erythroxylum coca* reference gene stability in a sample subset containing only leaf tissues (Buds, Leaf Stage I-III). Analysis was performed using the geNorm, BestKeeper and NormFinder algorithms.

Gene rank	geNorm (M*, V _n/n+1_)	BestKeeper (correlation coefficient, *r*)	NormFinder (stability value)
1	Ec10131/TPB1 (0.26)	Actin (0.869)	Ec6409 (0.176)
2		APT2 (0.868)	EF1α (0.264)
3	Ec6409 (0.30; 0.096)	Ec6409 (0.837)	Pp2aa3 (0.306)
4	Pp2aa3 (0.34; 0.089)	EF1α (0.805)	TPB1 (0.318)
5	Ec11142 (0.40; 0.088)	Pex4 (0.762)	Ec11142 (0.366)
6	EF1α (0.48; 0.100)	TPB1 (0.733)	Ec10131 (0.415)
7	Actin (0.68; 0.161)	Pp2aa3 (0.652)	Actin (0.642)
8	APT2 (0.78; 0.128)	Ec11142 (0.554)	APT2 (0.664)
9	Pex4 (0.90; 0.133)	Ec10131 (0.385)	Pex4 (0.815)

*M indicates stability values listed from most stable to least stable.

**Supplementary Table 2.** Ranking of *Erythroxylum coca* reference gene stability in a sample subset containing only matureorgans (Leaf stage III, Flowers, Roots, Stems). Analysis was performed using the geNorm, BestKeeper and NormFinder algorithms.

Gene rank	geNorm (M*, V _n/n+1_)	BestKeeper (correlation coefficient, *r*)	NormFinder (stability value)
1	Ec10131/6409 (0.17)	Pex4 (0.767)	EF1α (0.226)
2		APT2 (0.724)	Pp2aa3 (0.250)
3	Ec11142 (0.24; 0.088)	EF1α (0.693)	Ec11142 (0.273)
4	Pp2aa3 (0.27; 0.066)	Actin (0.576)	Ec6409 (0.332)
5	TPB1 (0.30; 0.064)	Pp2aa3 (0.538)	Ec10131 (0.395)
6	EF1α (0.45; 0.121)	TPB1 (0.452)	TPB1 (0.436)
7	Actin (0.61; 0.136)	Ec6409 (0.442)	Actin (0.522)
8	APT2 (0.74; 0.133)	Ec11142 (0.441)	APT2 (0.638)
9	Pex4 (0.97; 0.189)	Ec10131 (0.309)	Pex4 (1.170)

*M indicates stability values listed from most stable to least stable.
