# Supplementary material for: Selection and validation of reference genes for quantitative gene expression studies in Erythroxylum coca
Source: F1000Res. 2013 Feb 8;2:37. [Version 1] doi: 10.12688/f1000research.2-37.v1 (PMC3907153; doi:10.12688/f1000research.2-37.v1)
Supplement: Peer review history

# Peer review: Referee response for version 1

**Published:** 2013-02-15

**Authors:** Sarah O'Connor

**Affiliations:** 1 Biological Chemistry Department, John Innes Centre, Norwich, UK

I have read this submission. I believe that I have an appropriate level of expertise to confirm that it is of an acceptable scientific standard.

# Peer review: Referee response for version 1

**Published:** 2013-02-11

**Authors:** Sheila McCormick

**Affiliations:** 1 Plant Gene Expression Center, University of California, Berkeley, Berkeley, CA, USA

I have read this submission. I believe that I have an appropriate level of expertise to confirm that it is of an acceptable scientific standard.
